# Genome Variation Map of Domestic Qinghai-Tibet Plateau Yaks by SLAF-Seq Reveals Genetic Footprint during Artificial Selection

**DOI:** 10.3390/ani13182963

**Published:** 2023-09-19

**Authors:** Biao Li, Jinzeng Yang, Yili Liu, Mingfeng Jiang

**Affiliations:** 1 Key Laboratory of Qinghai-Tibetan Plateau Animal Genetic Resource Reservation and Utilization, Southwest Minzu University, Ministry of Education, Chengdu 610041, China; 18874028579@163.com (B.L.);; 2 Key Laboratory of Animal Science of National Ethnic Affairs Commission of China, Southwest Minzu University, Chengdu 610041, China; 3 Department of Human Nutrition, Food and Animal Sciences, University of Hawaii at Manoa, Honolulu, HI 96822, USA; jinzeng@hawaii.edu; 4 College of Animal Husbandry and Veterinary Medicine, Southwest Minzu University, Chengdu 610041, China

**Keywords:** yak domestication, population structure, natural selection

## Abstract

**Simple Summary:**

The yak is endemic to the Qinghai-Tibetan Plateau (QTP) and serves as an important livestock animal for Tibetan pastoral society. Phenotypic traits vary among domestic yaks. However, genomic characteristics, population structure, and the genetic relationship of domestic yaks with wild yaks have not been effectively characterized until now. In this study, six domestic populations were sequenced, and selective sweep regions involved in yak domestication were detected. Furthermore, divergence time for domestic and wild yaks was estimated. This study revealed new insight into selection for domestic and wild yaks.

**Abstract:**

The yak (*Bos grunniens*) was domesticated in the high-altitude QTP. Research about their genetic diversity and population structure is limited. In this study, we resequenced the genome of 494 domestic yaks using Specific-Locus Amplified Fragment Sequencing (SLAF-seq). The survey was conducted on six populations sampled from isolated locations in China in order to analyze their structure and genetic diversity. These six domestic populations were clearly grouped into two independent clusters, with Jinchuan, Changtai, and Jiulong showing a tight genetic relationship with the wild yak. Nerve development pathways were enriched with GO enrichment analysis of 334 domesticated genes. Major genomic regions associated with the differentiation of domestic yaks were detected. These findings provide preliminary information on the yak genome variability, useful to understand the genomic characteristics of different populations in QTP.

## 1. Introduction

The yak is endemic to the QTP and can live in harsh, challenging conditions, such as low oxygen supply, extreme cold, and scanty flora [1,2]. The yak was domesticated from the wild yak (*Bos mutus*) by the ancient Qiang people at the beginning of the Holocene [3,4,5]. It is an important livestock animal for Tibetan pastoral society. More than 14 million domestic yaks are currently preserved on the QTP. The yak provides basic resources such as meat, milk, and transportation for human living in high-altitude areas [6,7]. Domestic yaks in China vary in their intrinsic characteristics and are important genetic resources for yak husbandry worldwide. Therefore, understanding the genetic resource associated with domestic yak diversity would lay the foundation for further research and yak husbandry improvement.

Genome-wide analysis using resequencing technique gives the opportunity to detect genetic variation between wild and domestic plants and animals [8,9,10]. After variation map construction, SNPs and Indels can then be used as molecular markers to study genetic diversity and artificial selection for breeding. The identified favorable alleles will be directly applied for breeding [11,12,13,14]. The domestication process of yak species has been studied in recent years, using mitochondrial DNA [5,15], comparative genomics [16], and resequencing of native populations [3]. Assembling the yak genome identified an expansion in the yak gene families related to various biological pathways, such as sensory perception and metabolism, as well as the enrichment of protein domains involved in sensing the extracellular environment and hypoxic stress [16]. It gave genomic insight into the yak’s adaptation to high altitude. Population resequencing of wild and domestic yaks revealed genomic signals and candidate genes involved in yak domestication. The time of population expansion was calculated and was in accordance with human activity [16]. By analyzing mitochondrial D-loop sequences from 250 domesticated and 13 wild yaks, two separated maternal branches were identified, which diverged approximately 130,000–150,000 years ago [4]. Through continued comparisons of mitochondrial sequences among 405 domesticated yaks and 45 wild yaks, a third maternal branch was observed only consisting of wild yaks. Further, the effects of relaxed selection among domestic yaks due to the consequence of domestication was observed [17]. Although population structure and genetic diversity of domestic yaks have been previously analyzed using whole genome sequencing, the genomic signatures of yak breeds cannot be comprehensively uncovered due to the small number of analyzed samples [18]. Additionally, hybridization between domestic and wild yaks might have occurred during long-term artificial selection. Therefore, it is necessary to use a large-scale population collected from various domestic yaks to further study the yak genome. Moreover, the reference genome of domestic yaks was first reported in 2012 [16] and updated at the chromosome level twice using long-read sequencing technology [19,20]. The precise reference genome enabled genomic diversity, domestication, and population structure identification.

In this study, a total of 494 domestic yaks were sequenced, and 15 wild yaks were collected from published databases. Domestic yaks included Changtai, Jiulong, Jinchuan, Fenzui, Quanhei, and Fuluo populations, with Fenzui, Quanhei, and Fuluo belonging to the Maiwa population. Using large-scale resequencing, a genomic variation map was constructed. Population structure analysis revealed a genetic relationship between domestic and wild yaks. Genomic differentiation between and within wild and domestic yaks were analyzed, which uncovered selection in genes associated with yak domestication. The results of this study provided valuable genomic resources to study genomic characteristics of yak breeds/populations in QTP.

## 2. Material and Methods

### 2.1. Ethical Approval

This study was conducted following animal welfare requirements. The procedures approved for experiments were based on the recommendations of the Regulations for the Administration of Affairs Concerning Experimental Animals of China. The Institutional Animal Care and Use Committee of Southwest Minzu University approved all the animal experiments in this study (No. 16053).

### 2.2. Sample Collections, Sequencing, and SNP Calling

Sampling covered four different regions of QTP, including Jiulong (101 E, 29 N), Jinchuan (101 E, 30 N), Changtai (100 E, 31 N), and Hongyuan (101 E, 31 N). Blood samples were collected from 494 yaks belonging to six populations: Jiulong, Jinchuan, Changtai, Quanhei, Fenzui, and Fuluo. In detail, Quanhei (black body), Fenzui (black body color, but pink lips), and Fuluo (black at birth and grey as adults) are considered part of the Maiwa conserved population, which is reared at Hongyuan Yak Breeding Farm. Adult yaks of similar age, health status, and size were sampled. After the yak vein was pierced with a disposable blood sampler (China), blood was dropped into a 5 mL anticoagulant test tube and fully shaken. The samples were immediately put on ice and sent to Novogene Bioinformatics Technology Co., Ltd., for sequencing. DNA was extracted from individual blood samples with an TIANamp Blood Genomic DNA Kit (Tiangen Biotech Co., Ltd., Beijing, China). DNA purity and concentrations were measured with a Nanodrop 2000 spectrophotometer (Thermofisher Scientific, Franklin, MA, USA) based on OD260/280 values. The integrity of samples with an OD260/280 of 1.6–1.8 and a concentration of 50–1000 ng/µL were separated via 1% agarose gel electrophoresis (AGE) to ensure sample quality. Those samples that exhibited a single distinct AGE band were diluted to 50 ng/µL and stored at −80 °C. The specific-locus amplified fragment sequencing (SLAF) libraries were constructed following the procedure described by previous study, except that here two restriction enzymes, HaeIII (recognition site 5′-GG/CC-3′, New England Biolabs (NEB), Menlo Park, CA, USA)) and Hpy166II (5′-GTN/NAC-3′, NEB), were used to digest genomic DNA [21]. Pair-end libraries of digested genome were constructed and sequenced on an Illumina Hiseq 2000 Sequencer (Illumina, San Diego, CA, USA).

Low quality and adaptors containing reads were filtered using trimmomatic software [22]. The rest of the reads were mapped to yak reference (Bosgru_v3.0) [20] with BWA software [23]. Output files in SAM format were converted to bam files using Samtools software [24]. Then SNP calling was performed with GATK software [25]: (1) Duplicated reads were marked with MarkDuplicates command. (2) Individual samples were called using HaplotypeCaller command. (3) All samples were combined using GenomicsDBImport and SNP calling use GenotypeGVCFs. (4) SNPs were filtered with VariantFiltration weigh parameter of -filter “QD < 2.0” --filter-name “QD2” -filter “QUAL < 30.0” --filter-name “QUAL30” -filter “FS > 60.0” --filter-name “FS60” -filter “MQ < 40.0” --filter-name “MQ40. Finally, SNPs with a missing rate ≤ 0.5 and a minor allele frequency ≥ 0.05 were used for subsequent analysis. Furthermore, SnpEff software was used for annotating and predicting the effects of identified SNPs [26].

### 2.3. Phylogenetic Tree and Population Genetic Analyses

Phylogenetic tree was constructed using phylip software (https://evolution.genetics.washington.edu/phylip.html) (accessed on 20 January 2023): (1) Individual genetic distances were calculated using plink software [27] with parameter of --cluster --distance-matrix. (2) Neighbor function in phylip was applied to construct a Phylogenetic tree with the input of genetic distance file. Admixture software performed population structure analysis [28] with default parameters. Principal component analysis (PCA) was performed with gcta64 software [29]. Firstly, we obtained the genetic relationship matrix with the parameter “–make-grm”. Then, the top three principal components were estimated with the parameter “–pca2”.

### 2.4. Fst Estimation

We used the following formula to calculate Fst value between two populations:

$$\mathrm{Hs}=\frac{2xi\left(1-xi\right)+2yi\left(1-yi\right)}{2}$$

$$\mathrm{Ht}=\frac{2\left(xi+yi\right)}{2}\times \frac{\left(\left(1-xi\right)\left(1-yi\right)\right)}{2}$$

$$\mathrm{Fst}=1-\frac{Hs}{Ht},$$

where xi and yi refer to the frequency of the reference allele in populations 1 and 2. Firstly, Fst values for each SNP was calculated. Secondly, Fst values of SNPs in 100 Kb windows and 10 Kb step were analyzed. The top 5% windows were selected as significantly differentiated genomic regions. These regions were then merged if their genomic positions overlapped. Candidate genes in these differentiated regions were annotated and used for the functional enriched analysis.

### 2.5. Protein Functional Annotation and GO Enrichment

The longest transcript for each gene was used for functional annotation. The protein sequence was blasted against NR, COG, KOG, SWIFF databases using diamond software with cutoff of *p*-value ≤ 1 × 10^−5^ [30]. Next, GO annotation was performed. The protein sequence was used to predict GO function with Eggnog (v2.1.2) software [31]. R package clusterprofile was used to perform GO enrichment [32]. R package pheatmap (https://cran.r-project.org/web/packages/pheatmap/index.html) (accessed on 20 January 2023) was used to draw an enriched GO heatmap. GO terms with FDR ≤ 0.05 were selected.

### 2.6. π Ratio Estimates

To calculate nucleotide diversity in a given population, the following formula was used:

$$\pi =1-\sum_{i}^{n}(\frac{xi\left(xi-1\right)}{Si\left(Si-1\right)}),$$

where xi refer to the frequency of an allele in a given population, Si refers to the total observed alleles for *xi*, *i* = 1 or 2, which means the reference and alternative alleles, respectively. Nucleotide diversity was averaged in a 10 Kb window.

### 2.7. Identifying Selective Sweep Regions

To identify the regions under selective sweep between wild and domestic yaks, both Fst and π ratio analysis were applied, as this strategy can effectively identify sites under positive selection. Firstly, Fst values between wild and domestic yaks were calculated in 100 Kb windows with 10 Kb step. Secondly, π values were calculated for each wild and domestic population in 100 Kb windows with 10 Kb step. π ratio was calculated by comparing wild to domestic yaks in each corresponding genomic window. The top 5% genomic regions under selective sweep were selected. Third, the overlapping genomic regions resulting from Fst and π ratio analysis were considered as selective sweep regions for yak domestication.

### 2.8. Gene Flow and Divergence Time Analysis

Gene flow patterns between different populations were investigated using a maximum likelihood approach implemented in Treemix software with parameter of migration ranged from 0 to 4 [33]. The MCMCTree package in PAML 4.5 (http://abacus.gene.ucl.ac.uk/software/paml.html) (accessed on 20 January 2023) was used to estimate divergence times on the basis of the evolutionary tree and to further describe the differentiation time among different yak populations.

## 3. Results

### 3.1. Genome Variation Map of Yaks

**Figure 1:**
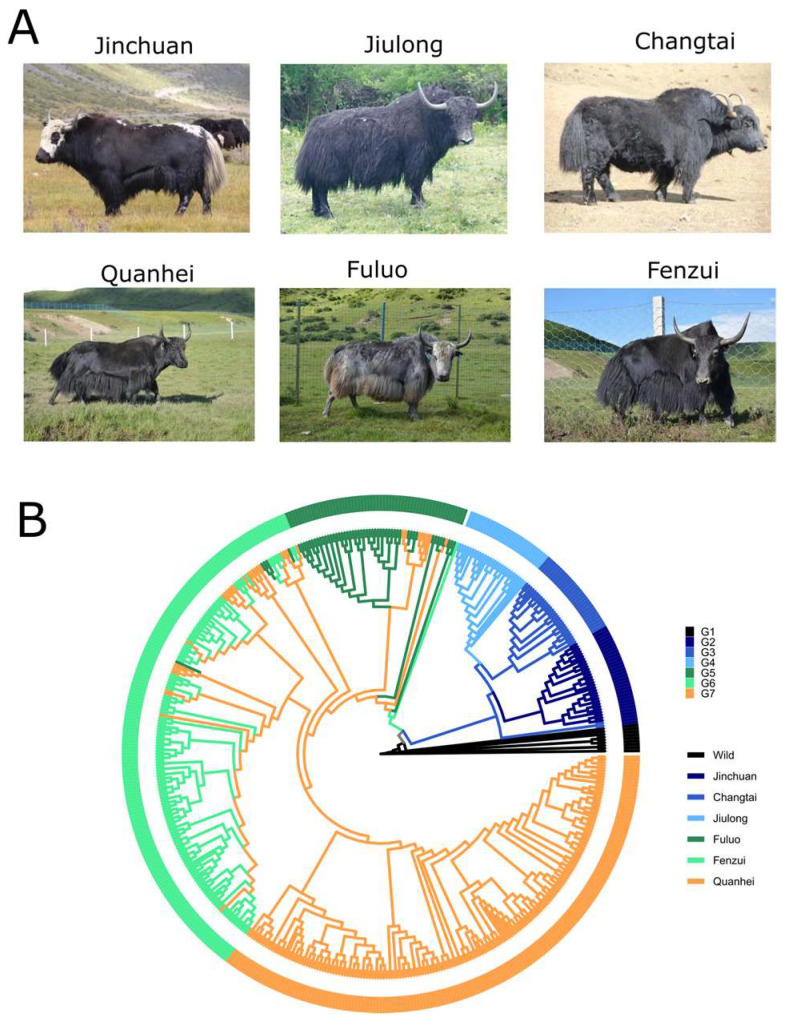
Domestic yaks and their phylogenetic tree. (**A**) Representatives of six different populations of domestic yaks; (**B**) Phylogenetic tree.

A total of 494 yaks representing six diverse populations in the QTP of China were collected for resequencing with SLAF-seq, including Jinchuan, Changtai, Jiuilong, and Maiwa (Fenzui, Quanhei, and Fuluo) (Figure 1A and Supplementary Table S1). These domestic yaks were widely distributed in the QTP. To detect genetic exchange and the relationship between domestic and wild yaks, genome sequencing data from 15 wild yaks were downloaded from NCBI for comparative analysis (Accession number: PRJNA285834). After reads filtering and SNP calling, a total 164,308 high-quality SNPs were obtained. These SNPs were mainly distributed in intergenic genomic regions, followed by intron, upstream and downstream of CDS, and exon (Supplementary Table S2). Among these SNPs, 441 SNPs were annotated as a missense mutation with SnpEff software. These SNPs were located in the functional domains of protein sequences and might be a valuable resource to study gene function of the yak genome.

Population-specific SNPs reflected the genetic diversity of the different populations. A total of 4402 private SNPs were identified for wild yak, followed by Maiwa, Fuluo, Changtai, and Jinchua yaks, which suggested a higher level of genetic diversity in wild yaks (Supplementary Table S3). A total of 2465 genes associated with these population-specific SNPs were involved in various biological pathways, such as olfactory and fatty acid biosynthetic processes (Supplementary Table S4). Therefore, these olfactory genes might be involved in olfactory tissue development between wild and domestic yaks. Also, we identified 1283~2312 specific SNPs for domestic yaks (Supplementary Table S4). For example, *DEFB* gene was associated with specific SNP identified in Quanhei yak and responsible for basic coat colors in cattle [34]. Therefore, these genes can be served as candidate genes to study the genetic diversity among different populations and used for further molecular breeding of domestic yak.

### 3.2. Phylogenetic Tree

To study the genetic relationship between wild and domestic yaks, a large-scale phylogenetic tree was generated using the neighbor-joining method with wild yak as the root (Figure 1B). The phylogenetic tree showed the individuals clustering mainly based on the population of belonging (Supplementary Table S5). Clusters G1 to G7 were dominatingly represented by Wild, Jinchuan, Chantai, Jiulong, Fuluo, Fenzui, and Quanhei yaks, respectively. For example, the G6 cluster included 139 Fenzui, 5 Fuluo, and 26 Quanhei yaks (Supplementary Table S5), belonging to populations reared in the same area. (Supplementary Table S5). Furthermore, we observed relatively close genetic relationship between wild yaks and Jinchuan, Changtai, and Jiulong yaks (Figure 1). This result indicated that Jinchuan, Changtai, Jiulong yaks might be hybridized with the wild yak for trait improvement.

### 3.3. Population Structure and Genetic Diversity

**Figure 2:**
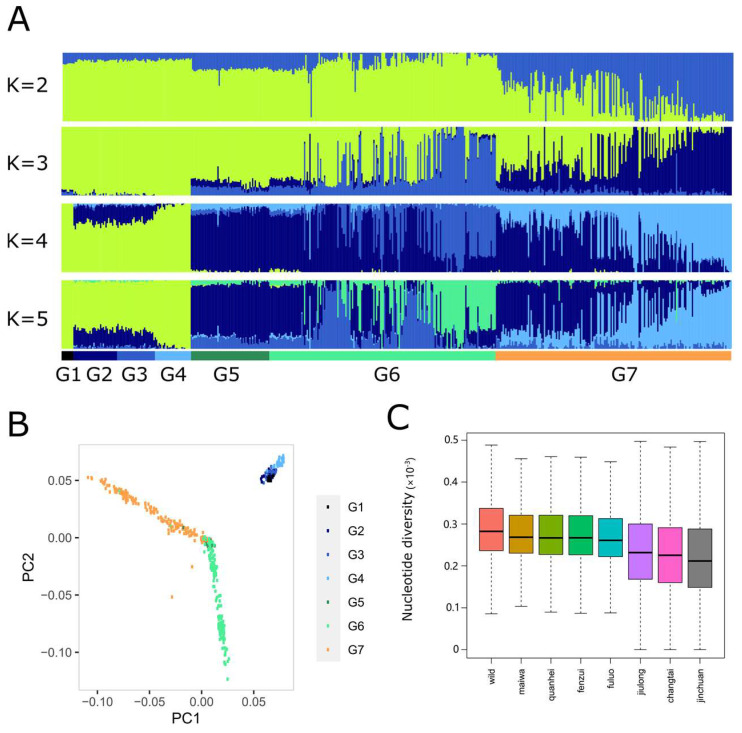
Population structure. (**A**) Structure analysis with different numbers of clusters (K = 2, 3, 4, and 5). The y axis quantifies clusters membership, and the x axis lists the different accessions; (**B**) PCA; (**C**) Nucleotide diversity of wild and domestic yaks.

Population structure analysis was performed to study the genetic composition of wild and domestic yaks (Figure 2A). Ancestry structure was studied from K = 2 to K = 5 (Figure 2A). When K = 2, domestic yaks, Jinchuan, Changtai, Jiulong, Fuluo, and Fenzui, were clustered with the wild yak, while Quanhei formed its own cluster (Figure 2A). From K = 3 to K = 4, Fuluo and Fenzui were both separated from wild yaks (Figure 2A). At K = 4, the Fenzui and Quanhei population structure was made up of 59.91% and 45.33% by the ancestry structure characterizing the Fuluo population. This result indicated that Fenzui and Quanhei might be selected from Fuluo yak (Figure 2A). The Jinchuan, Changtai, and Jiulong populations showed a tight genetic relationship with the wild yak and took up 70.16%, 70.79%, and 96.03% of the genetic composition of the wild yak, respectively (Figure 2A). However, these three populations contained 24.78%, 24.19%, and 3.17% genetic composition of Fuluo yak (Figure 2A), indicating its genetic contribution to the improvement of these three populations (Figure 2A).

The results of the population structure were resembled with the PCA output, with PC1 and PC2 explaining 20.76% and 17.72% of the genetic variation, respectively (Figure 2B). Jinchuan, Changtai, and Jiulong yaks showed a tight genetic relationship with the wild yak (Figure 2B). We found that PC1 mainly separated as (1) Quanhei, (2) Fenzui, and (3) Wild + Changtai + Jinchuan + Jiulong (Figure 2B).

We also calculated nucleotide diversity for wild and domestic yaks. As expected, the wild yak exhibited higher nucleotide diversity than the domestic yak (Figure 2C), suggesting that selection on the domestic yak decreased their genetic diversity. Jiulong, Changtai, and Jinchuan showed lower nucleotide diversity than domestic Maiwa yak, while Fenzui, Luofei, and Quanhei yaks exhibited moderate genetic diversity (Figure 2C). This result uncovered the genetic diversity of domestic yaks sequenced in this study.

### 3.4. Genome-Wide Selection and Genomic Differentiation Analysis

**Figure 3:**
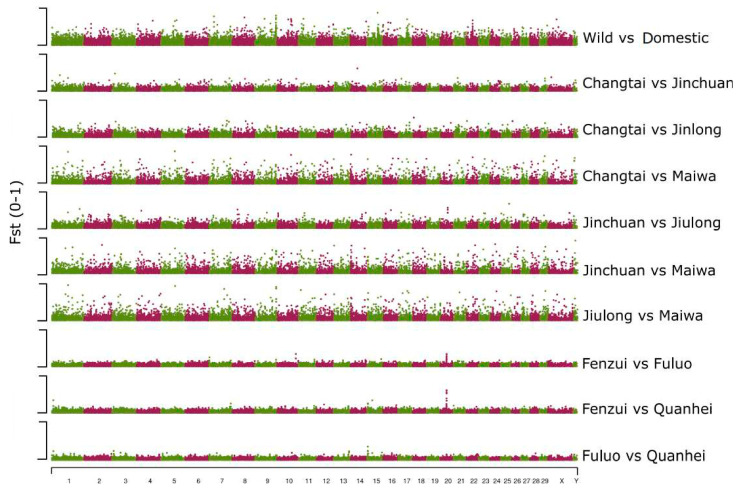
Fst analysis between and within wild and domestic yaks.

Wild yaks adapted to their natural environment while domestic yaks were artificially selected for human preferences, such as high milk production. To detect the selective footprints underlying wild and domestic yaks’ differentiation, we calculated pairwise Fst and π ratio (wild vs. domestic pairs) (Figure 3). Moreover, Fst analysis was used to detect differentiation among different populations of domestic yak. As expected, wild–domestic pairs showed the highest average Fst values (Figure 3). Fst analysis was also performed between Maiwa populations to identify genomic differentiation regions for each pair of different populations. Fenzui, Quanhei, and Fuluo exhibited relatively lower genetic differentiation than other domestic yaks (Figure 3), as they were reared in the same area and might be related to the same directional selection.

We detected 334 differentiated genes, which seem to be the target of artificial selections between wild and domestic yaks. Also, genomic differentiation regions between different domestic yaks included 614~1258 genes, covering a 36.2~76.2 Mb genome sequence (Supplementary Table S6). As a result of GO enrichment analysis, 4~129 GO terms were found to be enriched (Supplementary Figures S1–S10). For enriched GO terms between wild and domestic yaks, GO:0001964 (startle response) and GO:0098984 (neuron-to-neuron synapse) were overrepresented (Supplementary Figure S1), both of which are involved in brain and neuronal development. In the domestic yaks’ comparison, we found that GO terms related with nervous system development were also enriched in most of the population comparisons. This result suggests that nervous development might be involved in domestic differentiation.

As for the differentiation between different Maiwa populations, one significant genomic signal was detected on chromosome 20, spanning 33.62–34.275 Mb, by comparing Fenzui with Quanhei and Fuluo yaks (Figure 3). This genomic region might be responsible for phenotype differentiation between Fenzui and two other Maiwa populations. This region includes 19 genes, annotated as Myosin light chain kinase, Vacuolar protein sorting-associated protein, Dynein regulatory complex subunit, etc. One common, importantly genomic signal identified by comparing Maiwa with other three domestic yaks was located on chromosome 14, spanning 147,500~195,000 bp, although no gene was included in it (Figure 3).

### 3.5. Gene Flow and Divergence Time Analysis

**Figure 4:**
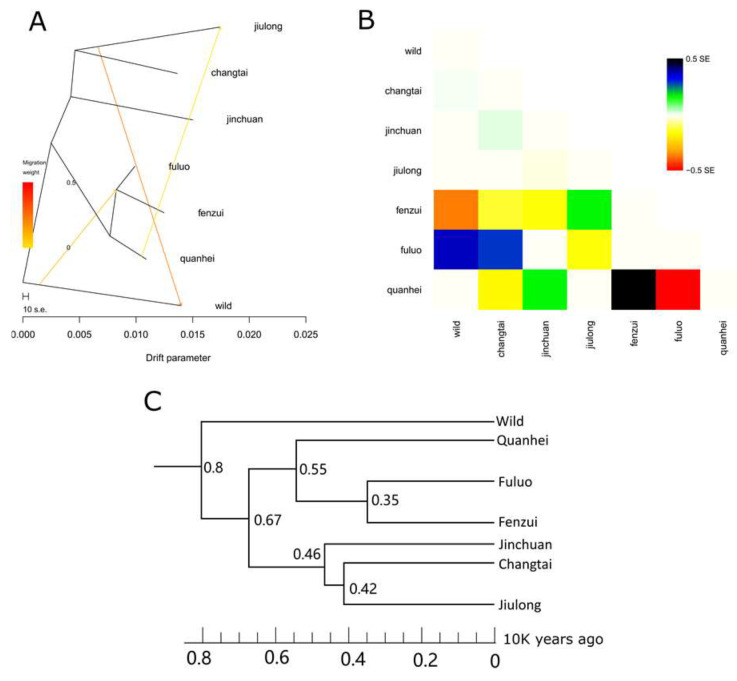
Gene flow and divergence time analysis. (**A**) Maximum likelihood tree with three migration events. The arrows (migration events) are colored according to their weight. The horizontal branch length is proportional to the degree of genetic drift in the branch; (**B**) Residual fit from the maximum likelihood tree in (**A**); (**C**) Divergence time analysis. Time is plotted along the *x*-axis.

Gene flow and divergence time analysis were analyzed with Treemix and SMC++ software, respectively. The migration parameter was tested from 0 to 4. At migration parameter 3, standard error (SE) decreased to near 0 (Figure 4A,B). Therefore, gene flow was examined at migration parameter 3. Results showed that migration was detected going from the wild yak to the ancestor of the Fuluo and Fenzui yaks (Figure 4A). Moreover, migration was detected going from Quanhei to Jiulong (Figure 4B). Furthermore, divergence time analysis for wild and domestic yaks was performed. Divergence time of wild and domestic yaks occurred about 8000 yr ago (Figure 4C). Then 6700 yr ago, two ancestors of domestic yaks were formed, that was (1) Quanhei, Fuluo, Fenzui; (2) Jinchuan, Changtai, Jiulong (Figure 4C).

## 4. Discussion

Genetic diversity is an important indicator for assessing population diversity. High genetic diversity helps species to adapt to changeable living environments. Previous studies demonstrated that, despite a long-term genetic bottleneck experienced by the wild yak, its genetic diversity was higher than that of the domestic yak [19]. However, another two studies found that domestic yaks showed higher genomic diversity than that of the wild yak [3,18]. In this study, we downloaded 15 wild yaks from the NCBI and compared genetic diversity between wild yaks and six populations of domestic yaks, which were sampled by our research team. The results showed that the genetic diversity of the wild yak was slightly higher than each population of domestic yak. However, when integrating all populations of domestic yak, the combination’s genetic diversity was higher than that of the wild yak. This result indicated a slight genetic bottleneck between wild and domestic yaks.

In previous studies, wild and domestic yaks were clearly divided into distinct lineages [18,19]. Indeed, phylogenetic tree analysis revealed two distinct lineages: Wild + Jinchuan + Changtia + Jiulong, and Maiwa populations. This result also was confirmed with population structure analysis. When K = 4, Jinchuan + Changtia + Jiulong maintained more than 70% of the wild ancestor’s composition than the Maiwa population, which was reviewed with PCA.

We observed a mixed genetic structure among the three Maiwa populations, indicating extensive genetic exchange. Fst analysis revealed a low level of genetic differentiation between Maiwa populations. Moreover, a major genomic region associated with the differentiation of Fenzui with Quanhei and Luofei was identified. This region, located on chromosome 20, might be involved in the specific phenotypic differentiation between Fenzui and other Maiwa populations.

Fst value was an indicator of genetic differentiation among populations. Fst showed weak (0–0.05), moderate (0.05–0.15), and significant (0.15–0.25) population differentiation. The paired populations were considered different when Fst exceeds 0.25 [35]. In previous study, using Y chromosome markers, Fst value between domestic and wild yaks was 0.178 [36], indicating paternal genetic differentiation. In this study, Fst value between wild and domestic yaks was 0.0471 on average, suggesting a moderate genetic differentiation between wild and domestic yaks.

Genome-wide analysis revealed regions under positive selection between wild and domestic yaks by integrating Fst and π ratio analysis. Especially, biological pathways involved in brain and neuronal development were enriched. Among these genes, *GRIN2B* encodes Glutamate receptor ionotropic that in humans is strongly associated with neuronal development [37]. *ANK2* encodes the Ankyrin repeat, which is involved in the transient increase in excitatory synapses during postnatal development [38]. *ANKS1B* encodes the CASK-interacting adaptor protein, functional in autism and neuropsychiatric diseases. Affected individuals present with a spectrum of neurodevelopmental phenotypes, including autism, attention-deficit hyperactivity disorder, and speech and motor deficits [39]. *NCAM2* encodes the neural cell adhesion molecule and is the second member of the NCAM family with a high expression in the nervous system, which controls important neuron-specific processes such as neuronal differentiation, synaptogenesis, and memory formation [40]. Olfactory development was also differentiated between wild and domestic yaks. *OR13J1*, *OR5M10*, and *OR2W3* functionals in olfactory development were under positive selection.

A total of six populations of domestic yak were sequenced. Among them, Fuluo, Fenuoz, and Quanhei are three conservation populations belonging to Maiwa yak. Fuluo yak has a grey coat color, while Fenzui and Quanhei yaks have a black coat color. Candidate genes responsible for their differentiation were identified. Among them, *KIT* gene encodes the mast/stem cell growth factor receptor, which is a cytokine receptor expressed on the surface of haematopoietic stem cells as well as other cell types [41]. This gene is located in the Dominant White locus and is confirmed to be responsible for color variation in pigs. Therefore, *KIT* genes might be responsible for coat color differentiation within the Maiwa population.

Divergence time between wild and domestic yaks was about 8000 yr ago, which was consistent with previously published research [42]. Divergence times between different yak populations were further tested in this study. The ancestors of Maiwa and other yaks diverged about 6700 yr ago.

## 5. Conclusions

In summary, a whole-genome resequencing strategy was used in this paper to explore genetic diversity, population structure, and genetic differentiation among six populations of domestic yak, which revealed major genomic loci associated with the differentiation of the domestic yak. Furthermore, selective sweep analysis revealed various biological pathways involved in yak domestication. Divergence time analysis estimated the parting time among and within domestic and wild yaks. This study provided new insight into differentiation among domestic yaks.

## Data Availability

Resequencing data were accessible at NCBI Bioproject (http://www.ncbi.nlm.nih.gov/bioproject, accessed on 1 May 2022) under accession numbers of PRJNA818054.

## Supplementary Materials

The following supporting information can be downloaded at: https://www.mdpi.com/article/10.3390/ani13182963/s1, Table S1. Sequencing information for 494 samples. Table S2. SNP distribution on genome. Table S3. Private SNP for wild and domestic yaks. Table S4. Private SNP associated genes. Table S5. Detailed information of clustered groups. Table S6. Private SNP associated genes. Figure S1. Go enrichment for Changtai vs Jinchuan. Figure S2. Go enrichment for Changtai vs Jiulong. Figure S3. Go enrichment for Changtai vs maiwa. Figure S4. Go enrichment for Fenzui vs Fuluo. Figure S5. Go enrichment for Fenzui vs Quanhei. Figure S6. Go enrichment for Fuluo vs Quanhei. Figure S7. Go enrichment for Jinchuan vs Jiulong. Figure S8. Go enrichment for Jinchuan vs Maiwa. Figure S9. Go enrichment for Jiulong vs Maiwa. Figure S10. Go enrichment for Wild vs Domestic.
