# Supplementary material for: Genome Variation Map of Domestic Qinghai-Tibet Plateau Yaks by SLAF-Seq Reveals Genetic Footprint during Artificial Selection
Source: Animals (Basel). 2023 Sep 19;13(18):2963. doi: 10.3390/ani13182963 (PMC10525144; doi:10.3390/ani13182963)
Supplement: Supplementary file 1 [file animals-13-02963-s001.zip › Supplementary material.pdf]

Supplementary Table S2 SNP distribution on genome

Genome feature	SNP number
Downstream	4004
Exon	1006
Intergenic	97727
Intron	56942
Upstream	4629

Supplementary Table S3 Private SNP for wild and domestic yaks

Subpopulation	Private SNP number
wild	4402
quanhei	2312
fenzui	2164
jiulong	2039
fuluo	1766
jinchuan	1283
chantai	1441

Supplementary Table S5 Detailed information of clustered groups

	Changtai	Fenzui	Fuluo	Jinchuan	Jiulong	Quanhei	Wild
G1	0	0	0	0	0	0	9
G2	2	0	0	30	0	0	0
G3	28	0	0	0	0	0	0
G4	0	0	0	0	28	0	0
G5	0	0	50	0	0	8	0
G6	0	139	5	0	0	26	0
G7	0	0	0	0	0	177	0

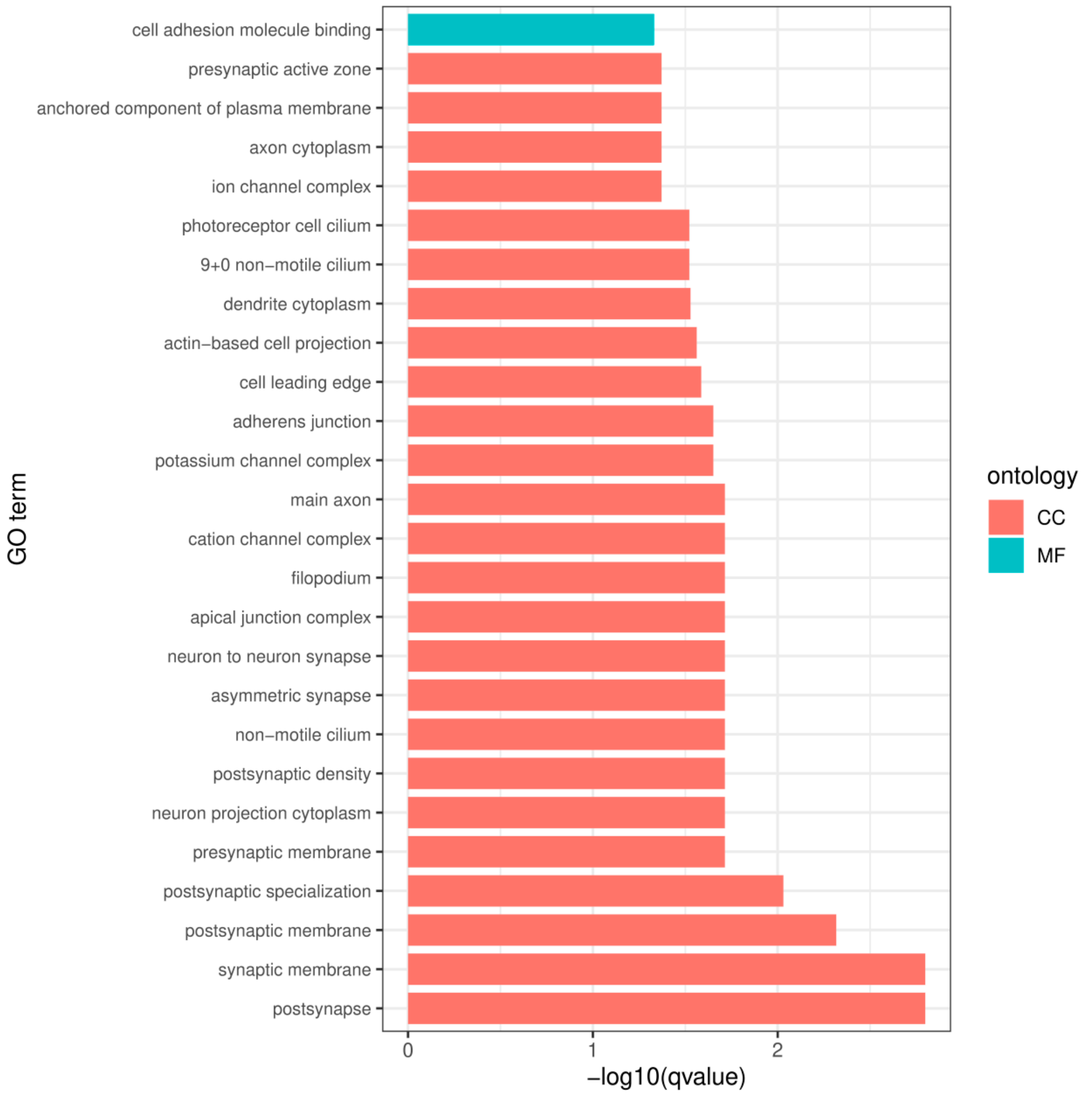

Supplementary Figure S1 Go enrichment for Changtai vs Jinchuan

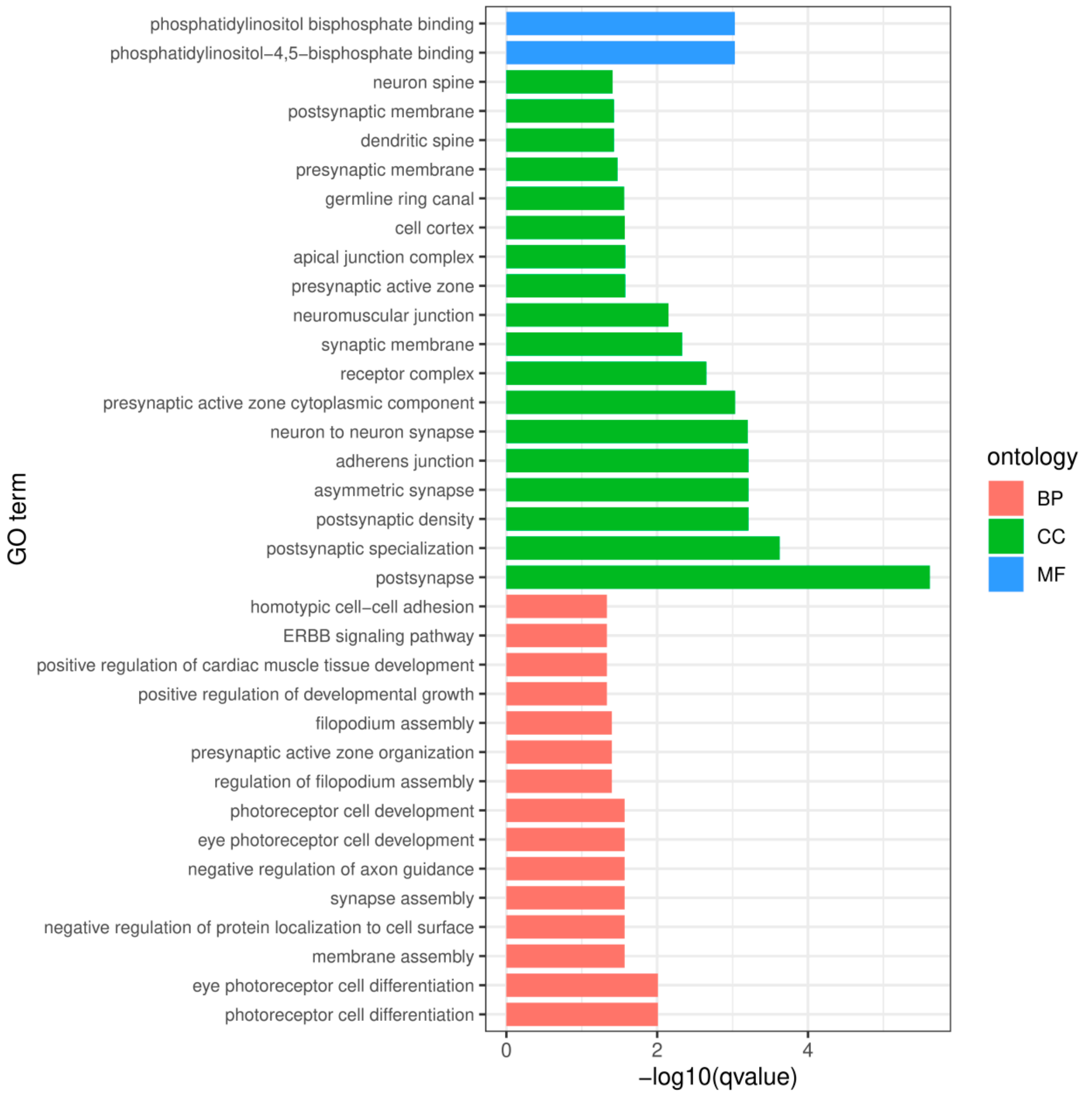

Supplementary Figure S2 Go enrichment for Changtai vs Jiulong

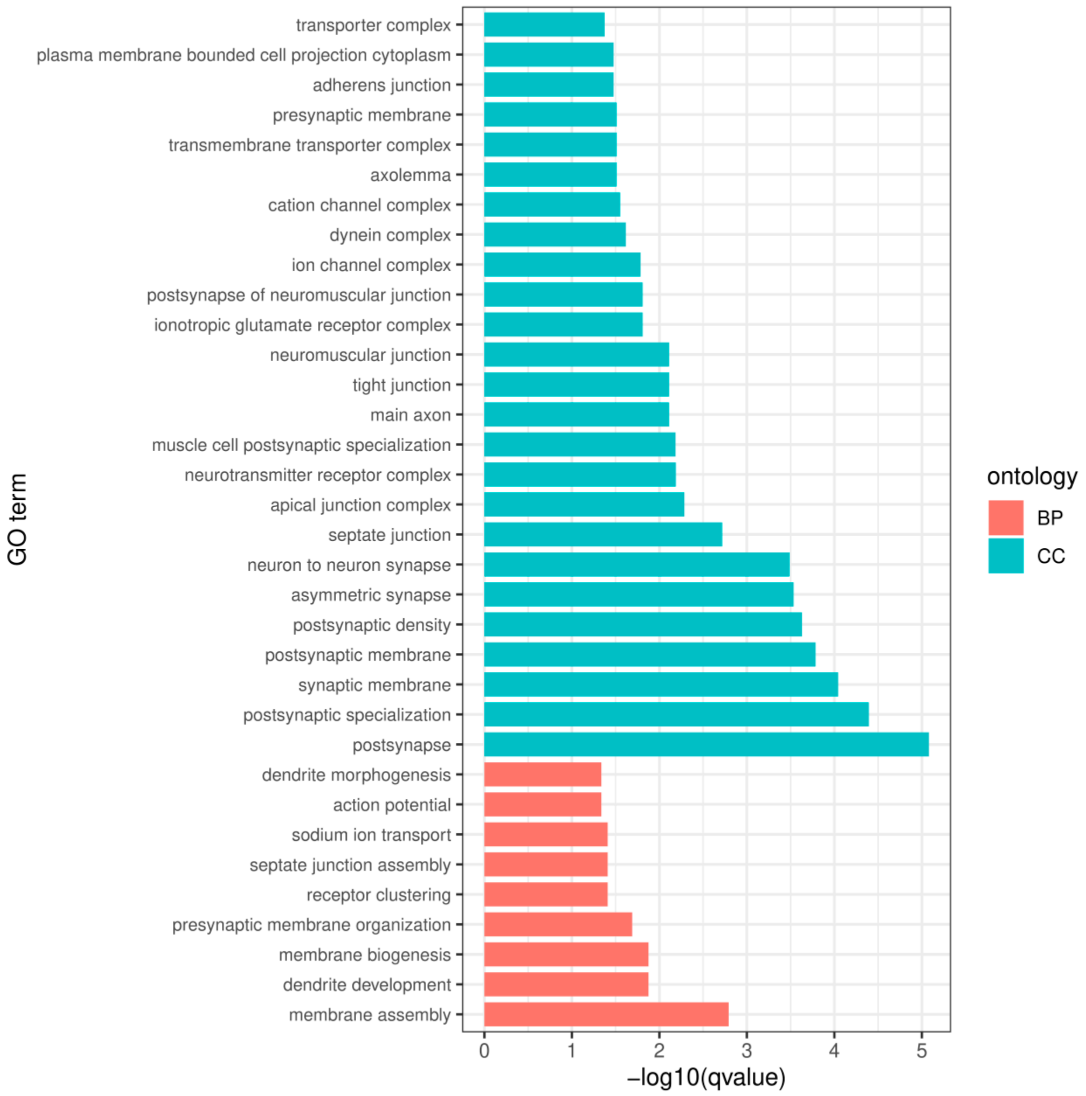

Supplementary Figure S3 Go enrichment for Changtai vs maiwa

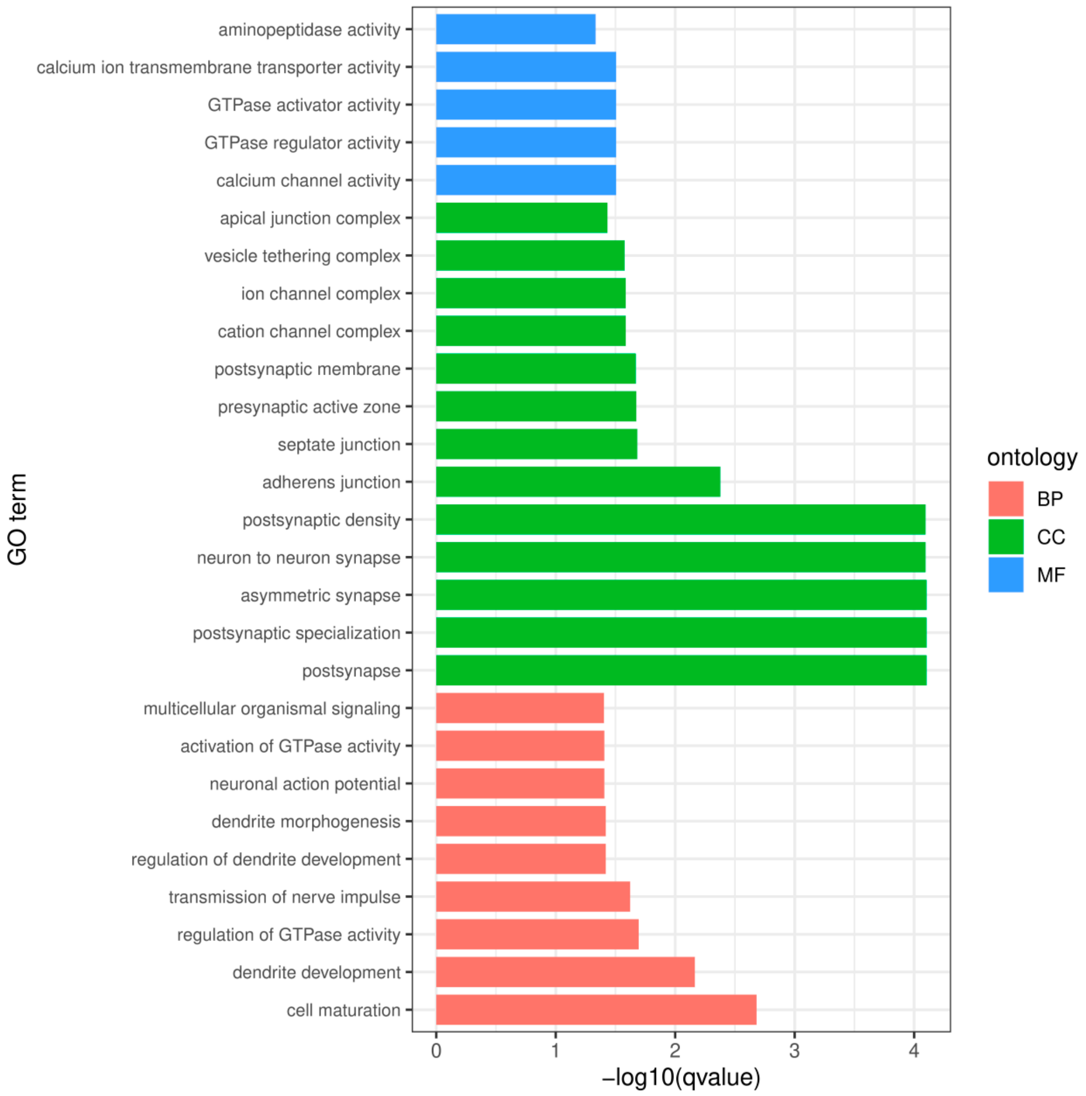

Supplementary Figure S4 Go enrichment for Fenzui vs Fuluo

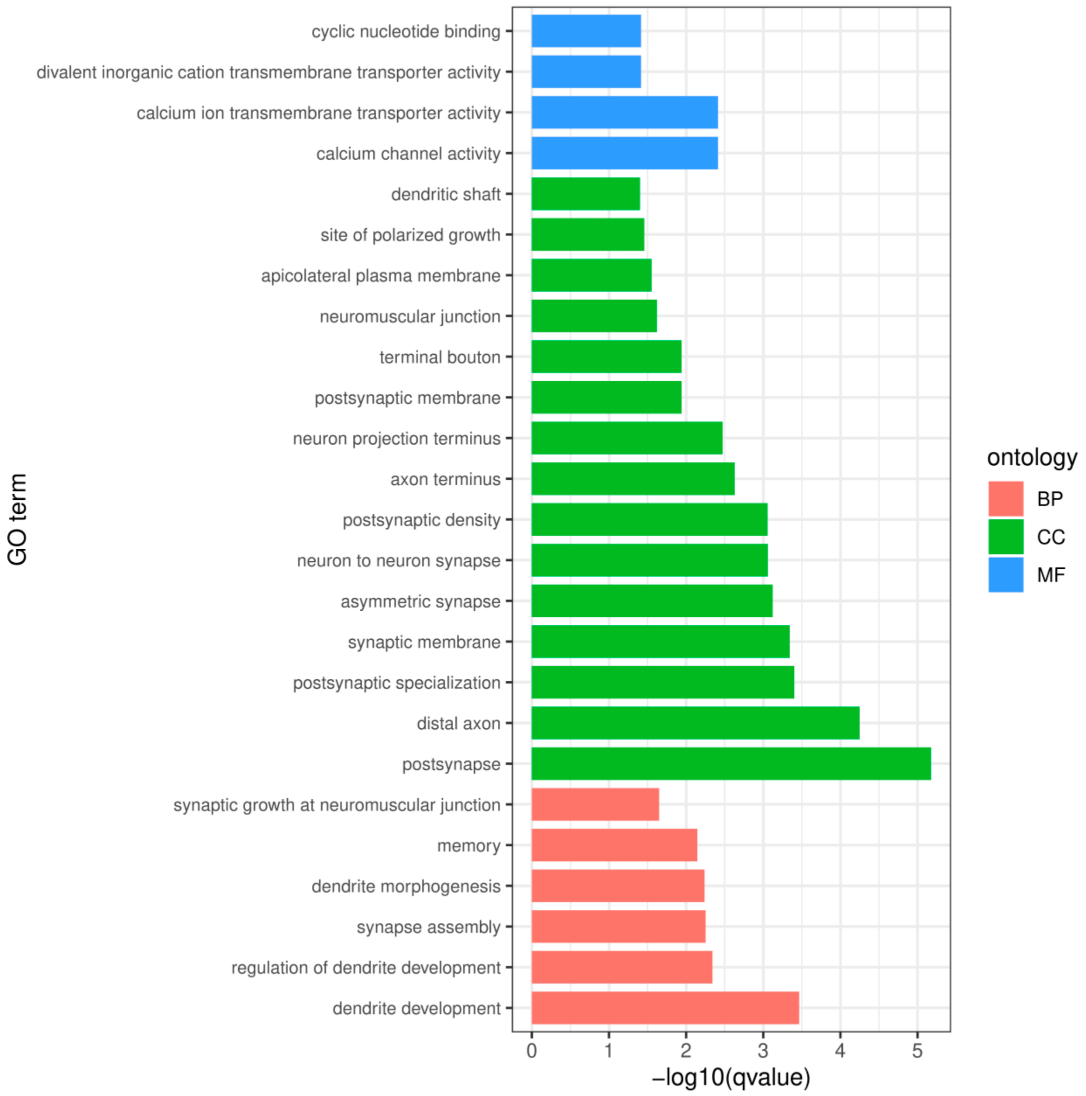

Supplementary Figure S5 Go enrichment for Fenzui vs Quanhei

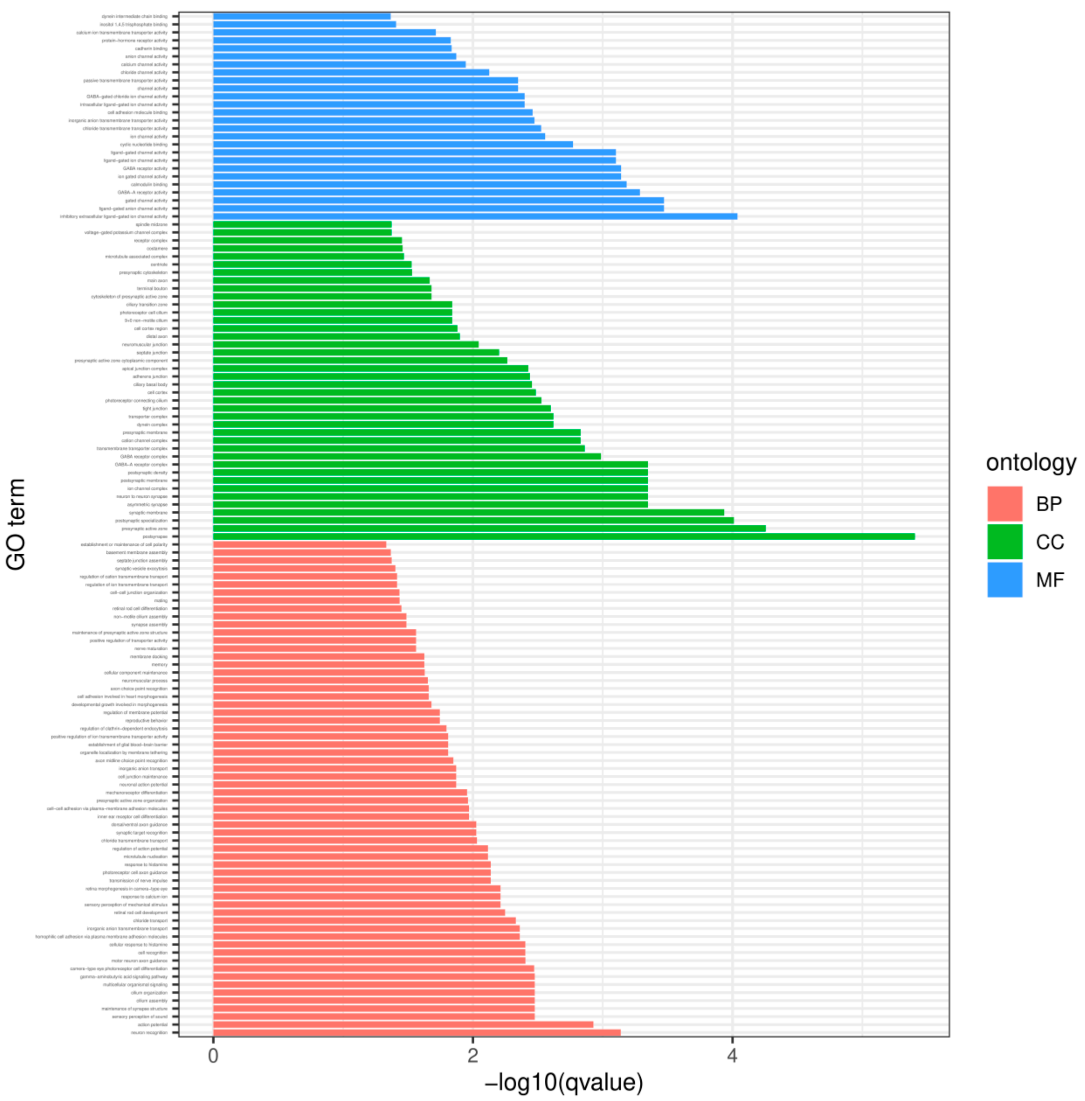

Supplementary Figure S6 Go enrichment for Fuluo vs Quanhei

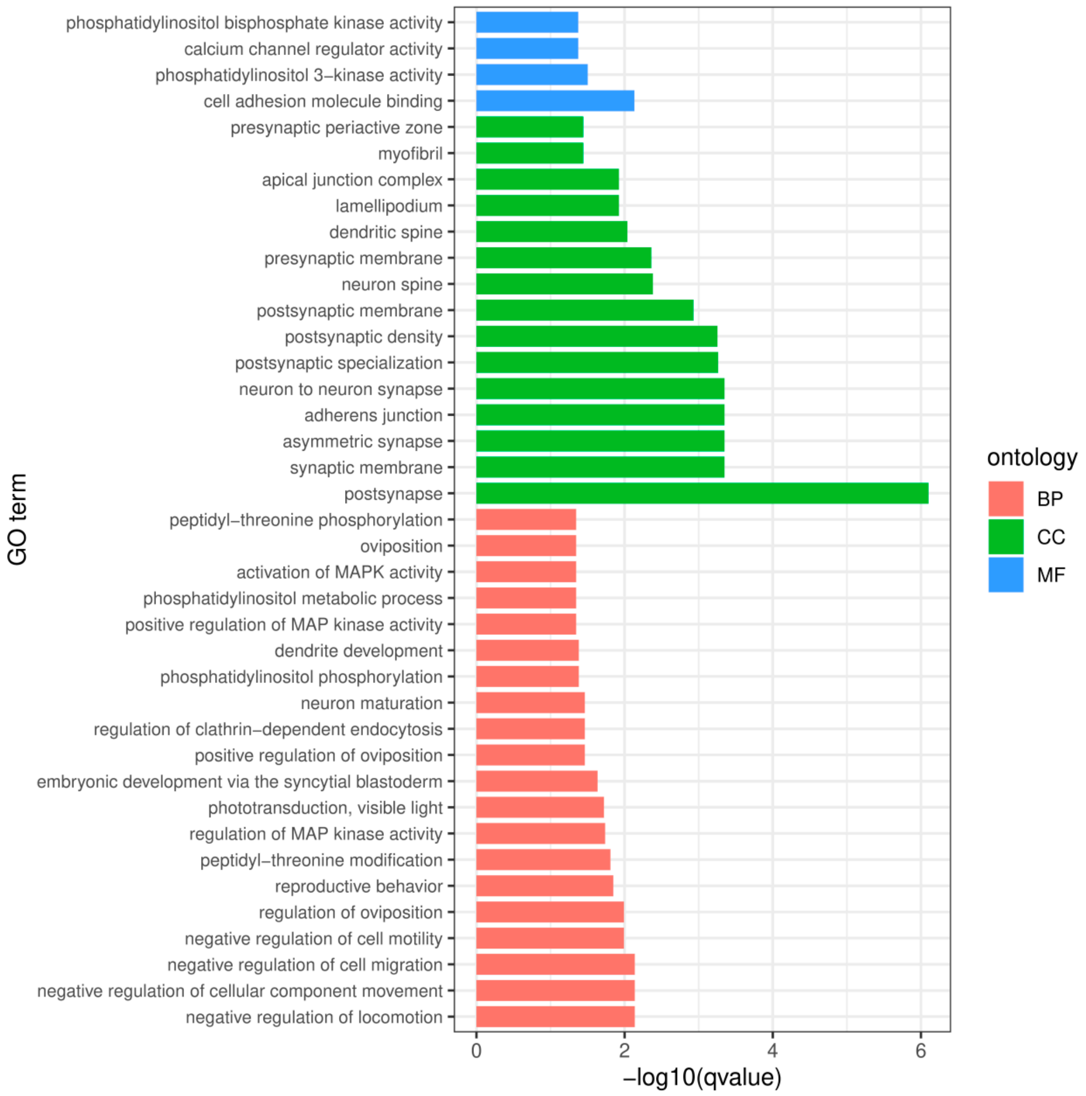

Supplementary Figure S7 Go enrichment for Jinchuan vs Jiulong

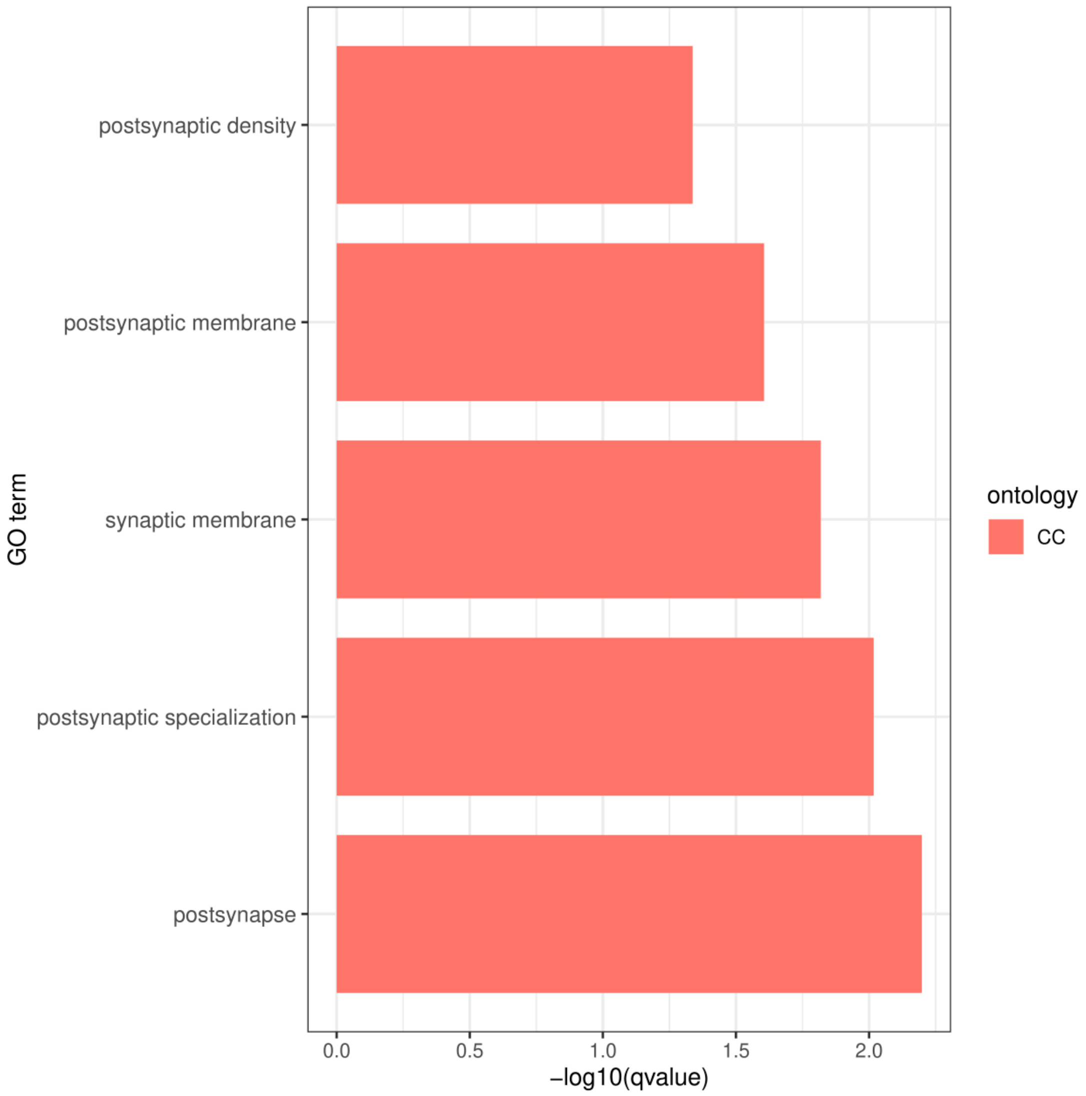

Supplementary Figure S8 Go enrichment for Jinchuan vs Maiwa

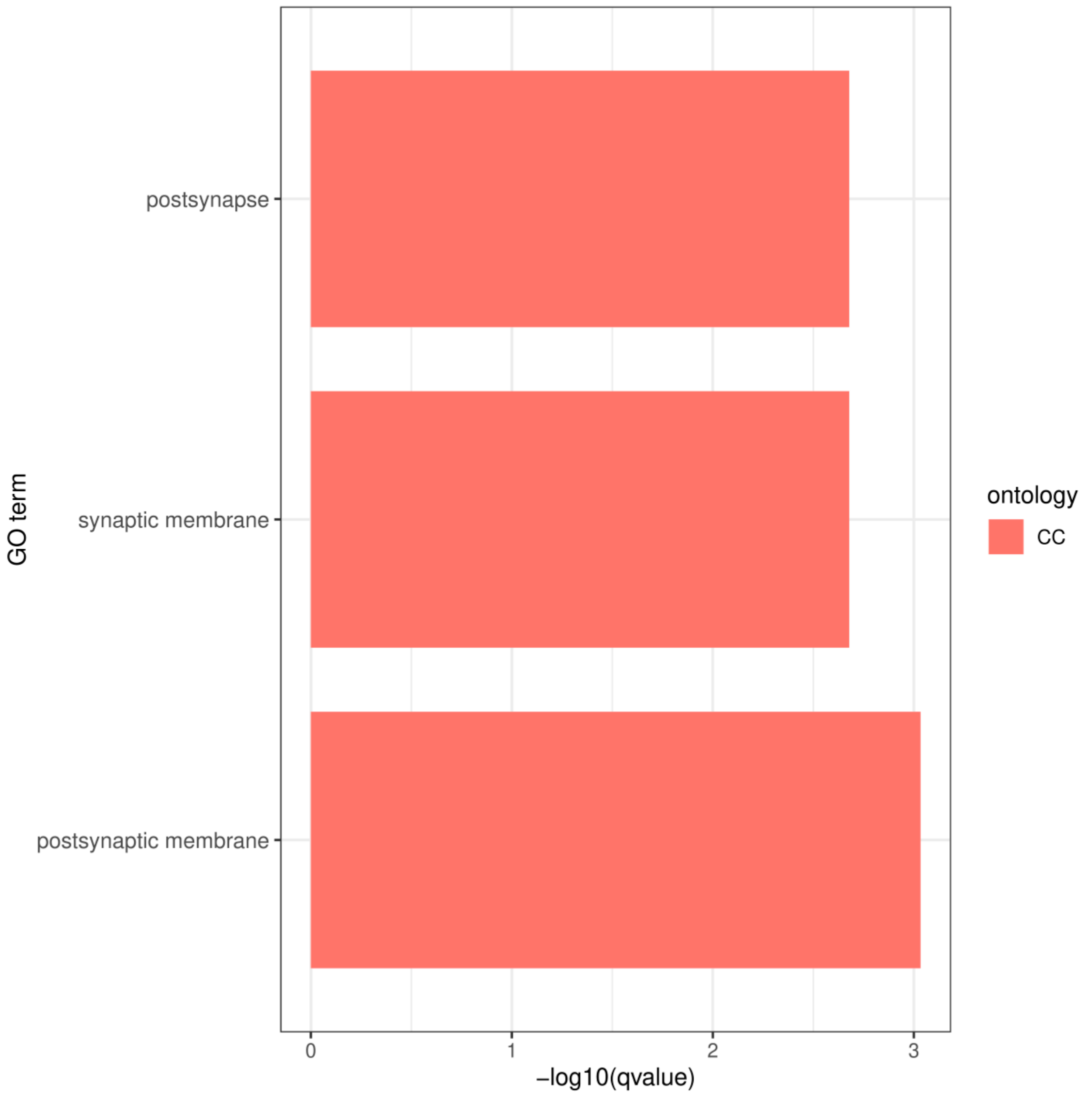

Supplementary Figure S9 Go enrichment for Jiulong vs Maiwa

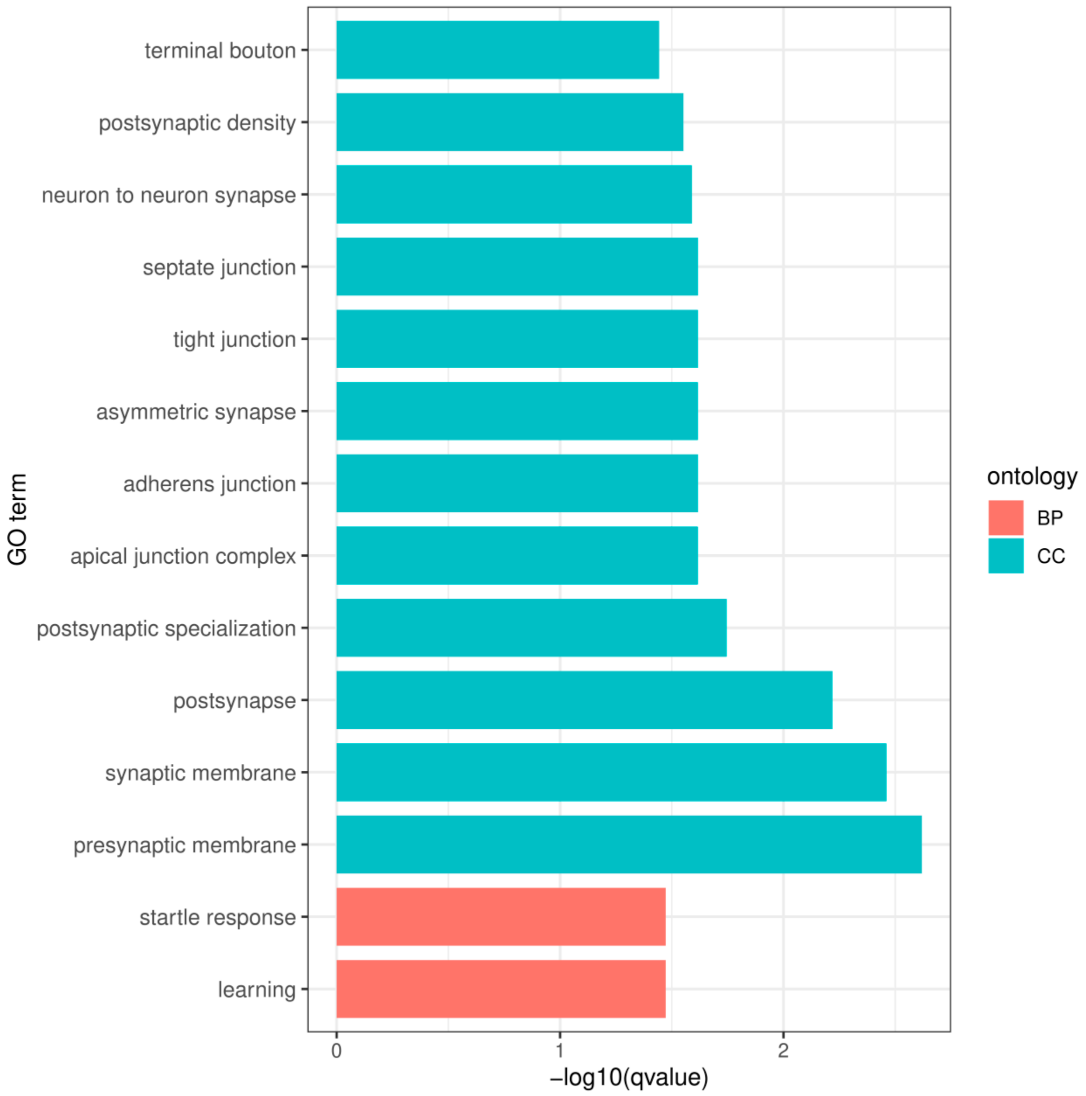

Supplementary Figure S10 Go enrichment for Wild vs Domestic
